# Experience of treatment of prosthetic valve endocarditis: a retrospective single-center cross-sectional study

**DOI:** 10.1590/1516-3180.2018.031150418

**Published:** 2018-07-21

**Authors:** Matheus de Oliveira Andrade, Gabriel Elias Salmen Raffoul, Murilo Teixeira Macedo, Claudia Neto Gonçalves Neves da Silva, Maria Aparecida Santos Teixeira, Sônia Jaciara Neto Pontes, Mauricio Daher, Claudio Ribeiro da Cunha, Fernando Antibas Atik

**Affiliations:** I Undergraduate Student, Universidade de Brasília (UnB), Brasília (DF), Brazil.; II Undergraduate Student, Universidade de Brasília (UnB), Brasília (DF), Brazil.; III MD. Cardiovascular Surgeon, Department of Cardiovascular Surgery, Instituto de Cardiologia do Distrito Federal (ICDF), Brasília (DF), Brazil.; IV MD, MSc. Infectiologist, Department of Infection Control, Instituto de Cardiologia do Distrito Federal (ICDF), Brasília (DF), Brazil.; V MD. Infectiologist, Department of Infection Control, Instituto de Cardiologia do Distrito Federal (ICDF), Brasília (DF), Brazil.; VI Nurse, Department of Infection Control, Instituto de Cardiologia do Distrito Federal (ICDF), Brasília (DF), Brazil.; VII MD, PhD. Anesthetist, Department of Cardiovascular Surgery, Instituto de Cardiologia do Distrito Federal (ICDF), Brasília (DF), Brazil.; VIII MD, PhD. Cardiovascular Surgeon, Department of Cardiovascular Surgery, Instituto de Cardiologia do Distrito Federal (ICDF), Brasília (DF), Brazil.; IX MD, PhD. Cardiovascular Surgeon and Head of the Department of Cardiovascular Surgery, Instituto de Cardiologia do Distrito Federal (ICDF), Brasília (DF), Brazil.

**Keywords:** Endocarditis, Heart valve prosthesis, Cardiac surgical procedures

## Abstract

**BACKGROUND:**

The aim of this study was to describe the experience of treatment of early prosthetic valve endocarditis at a heart center.

**DESIGN AND SETTING:**

Retrospective single-center study on data collected from electronic medical records covering the period from January 2009 to December 2015.

**METHODS:**

Over the study period, 1,557 consecutive valve operations were performed on adult patients. The study population comprised 32 patients (2%) who were diagnosed with prosthetic valve endocarditis within 12 months after the index surgery. Medical records were retrieved from electronic hospital records, retrospectively. Descriptive clinical, echocardiographic, microbiological and treatment-type data were used. Risk factors for early mortality were studied through univariate and multivariate analyses.

**RESULTS:**

The main clinical manifestation of infective endocarditis was fever, and this was present in all patients. Most of the prostheses were affected in the aortic position (40.6% of cases). The most commonly cultured microorganisms were *Staphylococcus epidermidis* and *Staphylococcus aureus*. Twenty-six patients (81.3%) underwent surgical treatment and six (18.7%) underwent exclusive clinical treatment. The prevalence of postoperative complications was 31.3% and hospital mortality occurred in seven cases (21.9%). The mortality rate was 50% among the patients who underwent medical treatment and 15.4% among those who underwent surgery. There were no independent risk factors for mortality.

**CONCLUSION:**

Prosthetic valve endocarditis is an infrequent complication of valve replacement. Surgical treatment has mortality rates compatible with the severity of patients’ conditions. Surgical indication should not be delayed when clinical treatment has been ineffective

## INTRODUCTION

Infective endocarditis is one of the most serious diseases that can affect heart valves. Despite technological developments that have been incorporated into new diagnostic methods and surgical techniques, the morbidity and mortality rates relating to infective endocarditis are still significant.^1^^,^^2^

Regarding native valve endocarditis, studies have shown that early surgical treatment is associated with lower complication rates and greater possibility of valve preservation, with obvious long-term benefits.^3^^,^^4^ However, early surgical treatment requires early diagnosis, which is not always possible. Presence of large valve destruction, ring invasion, abscesses, fistulas, distal embolization and multiorgan involvement may be frequent in cases of late diagnosis. The variability of the initial presentation to the surgeon gives rise to difficulties in patient management, and certainly impacts surgical results.^5^^,^^6^

Therefore, although the surgical indication for infective endocarditis has classic criteria that have been described in specific guidelines,^1^ there is controversy regarding the best moment to make the indication, when applicable. The patient’s clinical situation, the technical aspects of the operation and presence of extracardiac complications influence the therapeutic decision. Advanced age, important comorbidities and multiorgan dysfunction may be contraindications for surgical treatment.^5^

It is important to consider whether the infectious process reaches native valves or prostheses, since previously reported results have suggested that the evolution is worse in prosthetic valve endocarditis.^7^^,^^8^ Infective endocarditis of prosthetic valves presents a more complicated diagnosis, with less sensitivity to the Duke criteria.^9^ In addition, prosthetic valve infective endocarditis may be accompanied by perivalvular or myocardial abscess and valve dysfunction, thus presenting higher mortality than native valve infective endocarditis.^10^^,^^11^ However, there is a shortage of studies assessing operative outcomes from prosthetic valve endocarditis,^12^^,^^13^ and controversy remains regarding whether operative or conservative treatment should be indicated.

The objective of this study was to describe the experience of a cardiological center in managing early prosthetic valve endocarditis, regardless of the treatment instituted.

## METHODS

A retrospective single-center study was conducted through data-gathering from electronic medical records covering the period from January 2009 to December 2015. During this period, 1,557 consecutive valve operations were performed on adult patients, among whom 32 (2%) evolved with prosthetic valve endocarditis within 12 months after the index surgery. These patients constituted the population of our study. Patients who underwent surgery for correction or palliation of congenital heart diseases and native valve endocarditis were excluded.

The diagnosis of infective endocarditis was made in accordance with the modified Duke criteria.^14^ The classification of early prosthetic valve endocarditis was supported by the definition of the American Heart Association, which considers infectious involvement of the heart valve prosthesis within 12 months after the index surgery.^1^ In the cases evaluated here, once diagnostic suspicion had been established, cultures were collected and broad-spectrum empirical antimicrobial treatment was started while awaiting the culture results. In cases with a positive culture, antimicrobial treatment was guided by an antibiogram. The decision regarding surgical treatment and the best time to implement it was made following discussion within a team composed of a clinical cardiologist, a cardiovascular surgeon and an infectiologist. These decisions were made following wide-ranging discussion of individual cases.

Statistical analysis was performed using the JMP software (SAS version 9). Categorical variables were expressed as frequencies and percentages, and continuous variables as means and standard deviation. Multivariate analysis was performed by means of logistic regression to evaluate risk factors. The statistical significance level was taken to be 5%.

This study was approved by our institution’s research ethics committee (ICDF Ethics Committee; approved on August 29, 2013, under number 376261), in accordance with the Helsinki standards.

## RESULTS

### Clinical and echocardiographic characteristics

Over the study period, 1,557 consecutive valve operations were performed and 32 patients were diagnosed as having prosthetic valve endocarditis. Their mean age was 42.9 years and 16 of them (50%) were male. The clinical and echocardiographic characteristics of the patients are described in Table 1. The main cause associated with initial valve dysfunction was rheumatic heart disease, which was present in 28.1% of the cases.

**Table 1:** Patients’ clinical characteristics

Mean age (years)	42.9 ± 15.9
Age > 60 years	4 (12.5%)
Males	16 (50%)
Weight (kg)	63.9 ± 11.9
Height (cm)	165 ± 11.3
Body mass index (kg/m^2^)	23.6 ± 3.8
Systemic arterial hypertension	10 (31.3%)
Chronic kidney disease	6 (18.8%)
Previous stroke	3 (9.4%)
Rheumatic fever	9 (28.1%)
NYHA Functional Classification IV	4 (12.5%)
Diagnosis of infective endocarditis made within 30 days	18 (56.3%)

Endocarditis occurred within 30 days after the initial surgical procedure in 18 patients (56.3%), while 14 patients (43.7%) presented onset of infective endocarditis between one month and one year after the operation. Fever was the main clinical manifestation, and this was present in all patients.

The largest proportion of the prostheses were affected in the aortic position: 40.6%, comprising 11 cases in the aortic position alone and two cases in valved tubes. This was followed by cases affected in the mitral position (11; 34.4%) and in the mitroaortic position (6; 18.7%). The types of prostheses used in index surgery were biological in 18 cases (56.2%), followed by mechanical prostheses in 13 cases (40.6%) and a cryopreserved homograft in one patient. The prevalences of periprosthetic leakage, valve ring abscess and fistula were 34.4%, 9.4% and 3.1%, respectively.

### Microbiology

Blood culture positivity was low (6 patients; 18.8%). The percentage of patients undergoing antibiotic therapy was unknown. The following agents were identified: *Streptococcus sanguinis*, *Staphylococcus epidermidis*, *Staphylococcus aureus*, *Staphylococcus capitis* and *Clostridium* sp. Among the patients who underwent surgical treatment, six patients whose blood culture was negative had a positive valve prosthesis culture. The microorganisms of these cultures were: *Staphylococcus epidermidis*, *Staphylococcus aureus*, *Staphylococcus saprophyticus* and *Candida albicans* (Table 2).

**Table 2:** Blood and prosthetic valve cultures

Microorganism	Frequency
*Staphylococcus epidermidis*	4 (12.5%)
*Staphylococcus aureus*	3 (9.4%)
*Staphylococcus capitis*	1 (3.1%)
*Staphylococcus saprophyticus*	1 (3.1%)
*Streptococcus sanguinis*	1 (3.1%)
*Clostridium sp.*	1 (3.1%)
*Candida albicans*	1 (3.1%)
Negative cultures	20 (62.5%)

### Treatment

Out of the 32 patients diagnosed with prosthetic valve endocarditis, 26 (81.3%) underwent surgical treatment and 6 (18.7%) underwent exclusively clinical treatment. The decision on the type of therapy was based on the patients’ clinical conditions, the echocardiographic characteristics of the heart valves, possible complications and presence of important comorbidities.

Among the patients who were treated clinically, three individuals had clinical comorbidities that made the operation unfeasible (such as severe sepsis and cardiogenic shock) and evolved to death; two patients were receiving antimicrobial treatment for another condition (sepsis and wound infection) and improved; and one patient presented an initial operative indication but evolved with improvement after around one week of antibiotic therapy.

In the surgically treated group, the median length of time between diagnosis and surgery was 13.5 days. Regarding the initial valve operation (pre-endocarditis), the mean time taken for the cardiopulmonary bypass (cardiopulmonary bypass) procedure was 141.1 ± 54.1 minutes (median of 130.5 minutes). The time taken to perform the cardiopulmonary bypass was greater than 120 minutes in 19 patients (59.4%). The duration of intubation was over 48 hours in four patients (12.5%). Blood products were transfused in 21 patients (65.6%), with poly-transfusion in 11 cases. Postoperative hemodialysis was required in six patients (18.8%) (Table 3).

**Table 3:** Echocardiographic data before operation for infective endocarditis

Mean ejection fraction (EF) (%)	59.6 ± 14.3
Left ventricle dysfunction (EF < 50%)	6 (18.8%)
Right ventricle dysfunction	10 (31.3%)
Pulmonary hypertension (PASP > 40 mmHg)	12 (37.5%)
Presence of vegetative growth	30 (93.8%)
Presence of periprosthetic leakage	11 (34.4%)
Presence of fistula	1 (3.1%)
Presence of valve ring abscess	3 (9.4%)
Site of involvement	
Aortic	13 (40.6%)
Mitral	11 (34.4%)
Mitroaortic	6 (18,7%)
Other	2 (6.3%)

The prevalence of postoperative complications was 31.3%. Complete atrioventricular block occurred in four patients (12.5%), stroke in three (9.4%), sepsis in two (6.3%), limb amputation in two (6.3%) and acute myocardial infarction in one (3.1%).

### Mortality risk factors

Hospital mortality occurred in the cases of seven individuals (21.9%), thus representing 50% of the patients undergoing exclusively (Table 3) clinical treatment and 15.4% of the patients undergoing surgical treatment. Considering the echocardiographic and microbiological findings, along with the clinical factors associated with the initial operation, we did not identify any statistically significant risk factors for mortality through multivariate logistic regression analysis. This was probably due to the limited number of patients.

## DISCUSSION

The frequency of prosthetic valve endocarditis, considering total valve operations, was approximately 2%. This proportion is close to what was reported in the study by Pomerantzeff et al.,^13^ in which 28 patients presented prosthetic valve infective endocarditis out of a total of 1,512 valve operations (1.58%).

The diagnosis of infective endocarditis was established in accordance with the modified Duke criteria.^14^ Fever was the main sign that guided the suspicion of infective endocarditis in the postoperative period, and this was present in 100% of the cases. Other studies that assessed clinical manifestations of postoperative infective endocarditis have also highlighted fever as the most prevalent sign, present in 87% to 96% of the cases.^13^^,^^15^

Echocardiography plays an important role in making the diagnosis of infective endocarditis. Its findings may include perivalvular vegetations and abscesses, which may become more complicated through pseudoaneurysms and fistulization, or dehiscence of the prosthetic valve, which may result in perivalvular leakage.^16^ In our study, echocardiographic evaluation was fundamental for the diagnostic confirmation of infective endocarditis, and vegetative growths were detected in 93.8% of the cases. This frequency was higher than the 60% reported in another study.^13^

The aortic valve was the one most affected (40.6%), followed by the mitral valve (34.4%). This was concordant with previous studies that have mentioned that these valves are the ones predominantly affected by the infectious process.^13^^,^^17^ Mechanical and biological valves were similarly involved, which is also consistent with previous findings.^18^

Our study found that the positivity rate among the blood cultures was low (18.8%). Even considering prosthetic valve cultures, 56.3% of the patients did not present positive microbiological findings. Internationally, the estimated frequency of negative cultures in confirmed cases of infective endocarditis, considering both native valves and prostheses, is approximately 20%.^19^ Taking into account studies conducted in Brazil, the frequency of negative blood cultures ranges from 40% to 58%.^13^^,^^15^^,^^17^^,^^20^ Among these studies, only Pomerantzeff et al.^13^ specifically evaluated prosthesis endocarditis, and they reported that 50% of the blood cultures were negative (with the limitation of not describing prosthetic valve cultures).

The possible causes of high rates of negative blood cultures are inadequate culture techniques, uncultivated infectious agents and administration of antibiotics prior to sample collection for culture.^21^ Improvement of collection and culture techniques could increase the microbiological positivity indexes and optimize the clinical treatment targeted. In this context, the polymerase chain reaction (PCR) on valve tissue has also been highlighted as an effective method for microbiological confirmation, especially in cases of negative blood culture.^22^

In positive cultures, the most commonly isolated microorganisms were *Staphylococcus epidermidis* and *S. aureus*. In the literature, it has also been reported that early-stage prosthetic valve infective endocarditis (within one year after the index operation) is predominantly caused by coagulase-negative staphylococci or by *S. aureus.*^18^

In our series, the majority of the patients (about 80%) underwent operative treatment for prosthesis infective endocarditis. In an international multicenter cohort, the proportion of patients who underwent operations during the active phase of prosthetic valve infective endocarditis was approximately 50%.^12^

Despite the important role of surgery in the therapeutic management of infective endocarditis, only one randomized study of relatively small proportions has evaluated the role of valve operation in the treatment of endocarditis. That study demonstrated that the incidence of thromboembolic events was lower in the group that underwent surgery, but it was limited to only assessing patients with native valve infective endocarditis.^23^ There are no randomized studies of larger proportions that can provide unequivocal assurance regarding the benefit of early operation in treating infective endocarditis. Indications for early surgery are still based fundamentally on observational studies.

Surgical indication needs to be individualized based on a number of factors, such as age, clinical comorbidities, infectious agent, response to antimicrobial treatment, extent of vegetative growth, presence of perivalvular infection, presence of embolism or heart failure, and the surgeon’s experience.^1^^,^^6^ In the present study, six cases were managed exclusively with clinical treatment because of clinical comorbidities that made the operation unfeasible (such as severe sepsis and cardiogenic shock) or a good response after the initial antibiotic therapy.

Assessment of mortality through comparing surgical and clinical treatment is complicated by the fact that patients in a better clinical condition are more susceptible to undergoing surgery than those with early mortality (survival bias).^1^ The mortality rates of the exclusively clinical treatment and surgical treatment groups reported in this study (50% versus 15%) cannot be properly compared because of survival bias. A prospective multinational observational cohort showed that there was no difference in mortality between a group that underwent exclusively clinical treatment and a group that underwent early operation, after adjusting for survival bias.^12^

The prevalence of complications from the infective endocarditis operation was close to 30%, which was lower than the rate of 64% described in another study.^15^ We reported occurrences of complete atrioventricular block, thromboembolic events and sepsis, thus reaffirming the findings of previous studies.^13^ The operative mortality in our series was 15.4%, i.e. lower than that previously reported (27%),^12^ but this comparison is limited by the absence of a correction for the surgical mortality risk scores of the patients involved in the different studies.

Overall mortality (22%) was lower than the 30% mortality rate due to prosthetic valve endocarditis that has been reported in the literature from other countries.^5^ Brazilian studies have reported overall mortality rates ranging from 14% to 40%.^13^^,^^15^^,^^17^ The present study sought to establish echocardiographic findings and factors associated with the initial operation (Tables 1 and 2) that could characterize risk factors for mortality, but no statistically significant independent risk factors were identified. This was probably due to the small number of events.

Our study presented limitations regarding the retrospective collection, its single-center nature and its small sample. The relevance of the findings described here comes from the absence of randomized studies evaluating outcomes from operative treatment of prosthesis infective endocarditis, which means that management of patients affected by this condition is still based on observational studies.

## CONCLUSION

Prosthetic valve infective endocarditis is an infrequent complication of valve replacement operations, but it is associated with significant morbidity and mortality. Surgical treatment gives rise to mortality rates that are compatible with the severity of the patients’ conditions. The findings of our study reaffirm that surgical indications should not be delayed when clinical treatment is seen to be ineffective.
